# Models of microbiome evolution incorporating host and microbial selection

**DOI:** 10.1186/s40168-017-0343-x

**Published:** 2017-09-25

**Authors:** Qinglong Zeng, Steven Wu, Jeet Sukumaran, Allen Rodrigo

**Affiliations:** 1 Department of Biology, Duke University, Durham, NC USA; 2 Biodesign Institute, Arizona State University, Tempe, AZ USA; 3 Department of Ecology and Evolutionary Biology, University of Michigan, Ann Arbor, MI USA; 4 Research School of Biology, The Australian National University, Canberra, Australian Capital Territories Australia

**Keywords:** Microbiome, Selection, Diversity, Commensal and pathogen

## Abstract

**Background:**

Numerous empirical studies suggest that hosts and microbes exert reciprocal selective effects on their ecological partners. Nonetheless, we still lack an explicit framework to model the dynamics of both hosts and microbes under selection. In a previous study, we developed an agent-based forward-time computational framework to simulate the neutral evolution of host-associated microbial communities in a constant-sized, unstructured population of hosts. These neutral models allowed offspring to sample microbes randomly from parents and/or from the environment. Additionally, the environmental pool of available microbes was constituted by fixed and persistent microbial OTUs and by contributions from host individuals in the preceding generation.

**Methods:**

In this paper, we extend our neutral models to allow selection to operate on both hosts and microbes. We do this by constructing a phenome for each microbial OTU consisting of a sample of traits that influence host and microbial fitnesses independently. Microbial traits can influence the fitness of hosts (“host selection”) and the fitness of microbes (“trait-mediated microbial selection”). Additionally, the fitness effects of traits on microbes can be modified by their hosts (“host-mediated microbial selection”). We simulate the effects of these three types of selection, individually or in combination, on microbiome diversities and the fitnesses of hosts and microbes over several thousand generations of hosts.

**Results:**

We show that microbiome diversity is strongly influenced by selection acting on microbes. Selection acting on hosts only influences microbiome diversity when there is near-complete direct or indirect parental contribution to the microbiomes of offspring. Unsurprisingly, microbial fitness increases under microbial selection. Interestingly, when host selection operates, host fitness only increases under two conditions: (1) when there is a strong parental contribution to microbial communities or (2) in the absence of a strong parental contribution, when host-mediated selection acts on microbes concomitantly.

**Conclusions:**

We present a computational framework that integrates different selective processes acting on the evolution of microbiomes. Our framework demonstrates that selection acting on microbes can have a strong effect on microbial diversities and fitnesses, whereas selection on hosts can have weaker outcomes.

**Electronic supplementary material:**

The online version of this article (10.1186/s40168-017-0343-x) contains supplementary material, which is available to authorized users.

## Background

In a recent paper [1], we developed an agent-based framework to model the evolution of host-associated microbial communities (i.e., microbiomes), in the absence of any fitness costs or benefits to hosts or microbes, and subject only to the stochasticity of sampling. Other studies [2–5] have also explored how microbial communities are assembled in the absence of selective effects. Neutral theories assume that species are ecologically and functionally equivalent and that stochastic processes are the main factors shaping species’ distributions and community structure [6–8]. In at least some instances, neutral models are able to reconstruct and predict species abundance distributions in many natural microbial communities [9–11].

Nonetheless, there is considerable evidence that both microbes and hosts contribute reciprocally to the fitness of their ecological partners: Li and Ma [5], in a recent analysis of human microbiomes, concluded that less than 1% of human microbial communities are present because of neutral processes. Host-associated microbes often benefit from a protected and nutrient-rich environment. We know, for instance, that the stable temperature and pH within the human gut allows the colonization and cultivation of some microbial species but not others [12, 13] and the inability to utilize gut specific substrates can restrict the growth of some microbes [14, 15]. Conversely, studies have demonstrated associations between the presence or absence of one or more microbial taxa and host health and fitness. Gut bacteria assist their hosts with digestion in many vertebrate and invertebrate species [16–18] and rhizobia, which occupy the root nodules in legumes, supply ammonia for fixing nitrogen [19, 20], and in humans, microbiome composition has also been associated with a variety of physical and mental disorders, including inflammatory bowel disease [21–23], obesity [17, 24], allergic responses [25], anxiety [26], and autism [27, 28]. Biologically, then, it is reasonable to extend the neutral model of microbial evolution to allow selective effects: conceivably, hosts with beneficial microbes are more likely to succeed in competition with hosts that have fewer such microbes; it is also likely that microbes that are able to withstand the selective filters imposed by environments within hosts, and host antimicrobial defenses, are able to persist within individual hosts.

Li and Ma’s [5] analysis of human microbiome communities, based on work that comes directly from the neutral theory of biodiversity [29], suggests how we may test the null hypothesis of neutral microbial community composition, but does not provide a framework for uncovering patterns of diversity that may result from various selective processes. In this paper, we take on the task of modeling the simplest of these selective processes by extending our earlier agent-based framework. As in our earlier paper, we remind readers that the simplicity of these models in no way reflects a belief that natural systems are simple; rather, it is an approach that reveals what outcomes are possible in the absence of the added complexity that nature often engenders. In doing so, it offers a means for researchers to discern when the data are consistent with simple processes and, in so doing, resist the natural temptation to add what may appear to be intuitively obvious (but unnecessary) biological complexity.

Superficially, we may think that we should be able to model changes in the frequencies of microbial taxa in ways that are analogous to alleles or traits under selection. However, microbes are not necessarily transmitted vertically from parent to offspring in the same way that genes are. In our neutral models of microbiome evolution, hosts acquire their microbiomes from their parents and/or the environment, and it seems appropriate to apply the same mechanisms of microbial acquisition when extending these models to include effects of selection. To construct the environmental complement of microbes, therefore, we allow a component of environmental microbiome that remains constant over evolutionary time, as well as a component that consists of microbial contributions from each successive parental generation. As with our neutral models, our forward simulations are agent-based, with constant numbers of hosts across discrete generations.

To investigate the effects of selection, we allow microbes to influence the reproductive success of the host (i.e., “host selection”); we also assign to microbial OTUs different fitness values, by either allowing the host to influence the microbe’s ability to survive within it (i.e., “host-mediated microbial selection”) or by assigning traits that confer a selective advantage regardless of host (i.e., “trait-mediated microbial selection”). Whereas it is possible to model these selective effects by defining fitness contributions as a random variable drawn from an appropriate probability distribution, we have chosen a different approach. We model these selective effects by constructing a “phenome” for each microbial taxon, that is, a complement of traits, each of which contributes a positive, neutral, or negative effect to the host and, independently, a positive, neutral, or negative effect to the microbe itself, either influenced by the host or not. We have adopted a phenome-based approach because of its generalizability and flexibility: in this paper, it provides us with a good way of modulating fitness, and in the future, we will use this approach to study lateral gene transfer amongst microbes [30–33], as well as epistasis [34–36].

Our results indicate that selective effects that act on microbial fitness exhibit a significant depressive effect on microbiome diversities, regardless of whether these effects are host-mediated or trait-mediated. In contrast, microbes that collectively exert an effect on host fitness have only a weak effect on microbiome diversities, unless there is high parental contribution from one generation to the next. Our models can also account for the persistence of microbes within hosts that have no effect on host fitness (i.e., “commensal” microbes) and the persistence of microbes that have negative fitness effects on hosts (i.e., “pathogenic” microbes) within populations. Finally, if we examine the impact of the different selective effects on changes in average host or microbial fitness over evolutionary time, we see that when microbes are subject to either host- or trait-mediated selection, average microbial fitness increases. Increasing average host fitness requires at least two conditions: (1) microbes influence host survival and/or reproductive success, and (2) microbes are transmitted parentally/socially or microbes are themselves subject to host-mediated selection.

### Model

We recently proposed agent-based neutral models of microbiome evolution [1]. The basic framework of our neutral models incorporates a Wright-Fisher genealogical model of hosts with discrete generations; this allows us to investigate the dynamics of microbiomes on an evolutionary timescale in which some host lineages are lost and others amplified stochastically. We also incorporated neutral models of microbiome acquisition and environmental microbial community assembly that rely exclusively on random sampling from microbial communities. The acquisition of microbiomes by hosts has been modeled according to a “mixed-acquisition” (MA_*x*_) process, in which hosts acquire some percentage, *x*%, of their microbiomes from their parents and (100 − *x*)% from the environment. In our model, when *x* = 0, all microbes are acquired from the environment, and we refer to this as pure environmental acquisition (EA); conversely, when *x* = 100, all microbes are acquired vertically from parents (pure parental acquisition or PA). These models of acquisition also require us to specify mechanisms for how the environment is constituted. Analogously, we construct a “mixed environment”(ME_*y*_) model, where the environmental microbial pool contains a percentage, *y*%, from the community obtained by pooling all parental microbiomes, and (100 − *y*)% of microbes from a fixed and unchanging environmental pool. Since we are working with discrete generations of hosts, this allows us to model the phenomenon whereby each preceding generation of hosts alters the microbes available for recruitment by the subsequent generation. When *y* = 0, all microbes that are available in the environment come from a fixed pool (i.e., a pure fixed environment or FE); when *y* = 100, all microbes in the environment are derived from the collective microbial content of the previous generation (i.e., the pure pooled environment or PE). The basic neutral models are consistent with the neutral theory [6] in which there are no differences amongst hosts or microbes that can affect microbial community composition.

**Fig. 1:**
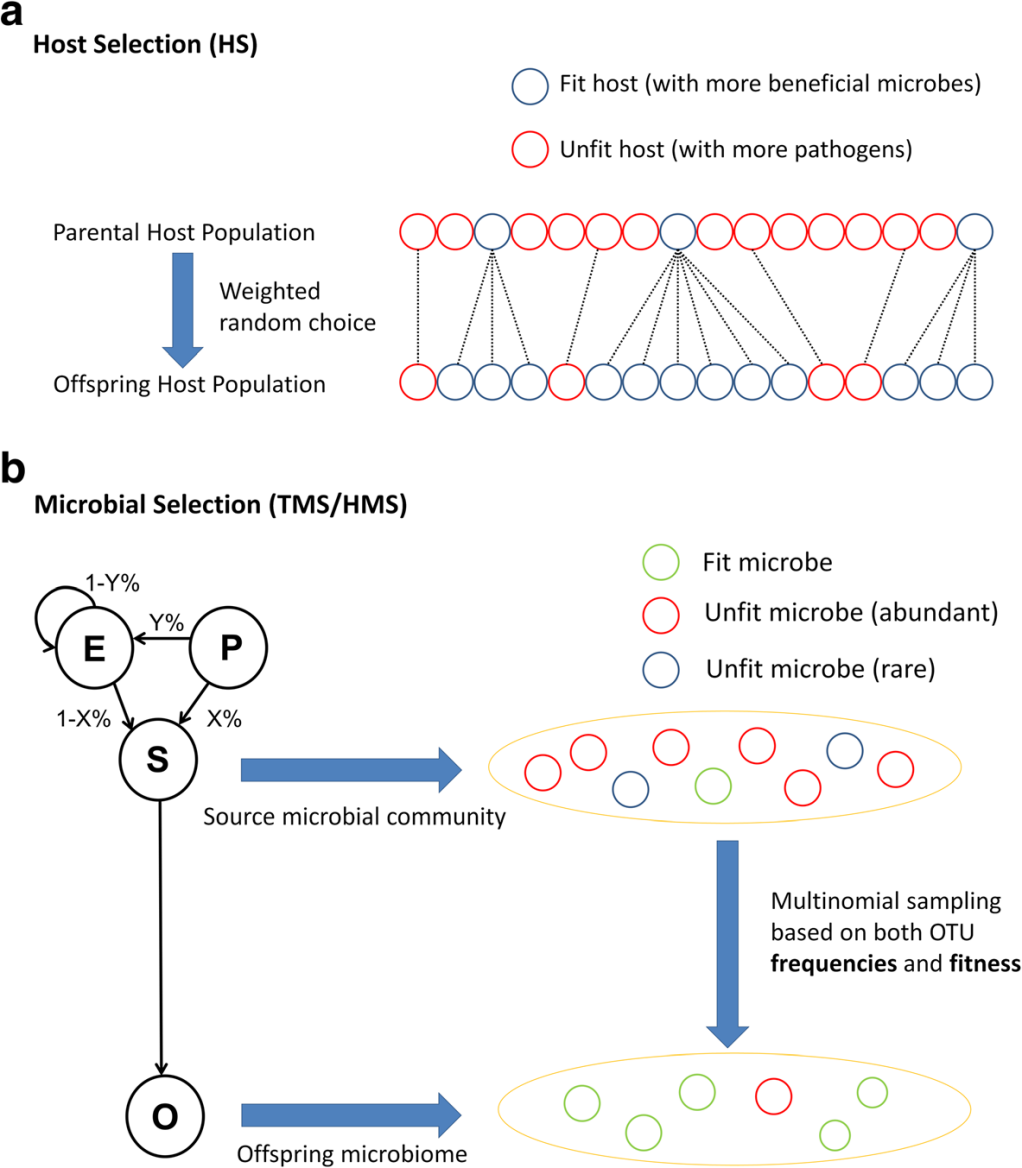
Host reproduction and microbial community assembly under models of selection. **a** A schematic showing the action of host selection on numbers of offspring. Each small circle represents a host with the first row representing the parental host population and the second row representing the offspring host population. The color indicates the fitness status of hosts which is completely determined by the microbes hosts carry (blue, fit; red, unfit), and the dotted lines indicate the parent-offspring relationship between generations. Fitter hosts produce relatively more offspring, weighted by parental fitness. **b** The relative abundance of microbial OTUs in host offspring, taking account of microbial fitness values. The letters “P,” “E,” “S,” and “O” stand for parental, environmental, source, and offspring microbial communities, respectively. The values of *Y*% and *X*% represent the percentage of host contribution under mixed environment (ME), and the percentage of parental inheritance under mixed acquisition (MA), respectively. Two yellow ellipses represent the source community (either parental, environmental, or mixed microbiomes depending on the value of *X*), and the offspring microbiome with a blue arrow indicating how microbes are sampled. Each small circle stands for a microbe with its color indicating the fitness status (green, fit; red, unfit but abundant in source community; blue, unfit and rare in source community). Fitter microbes and abundant microbes are more likely to be acquired by offspring

**Fig. 2:**
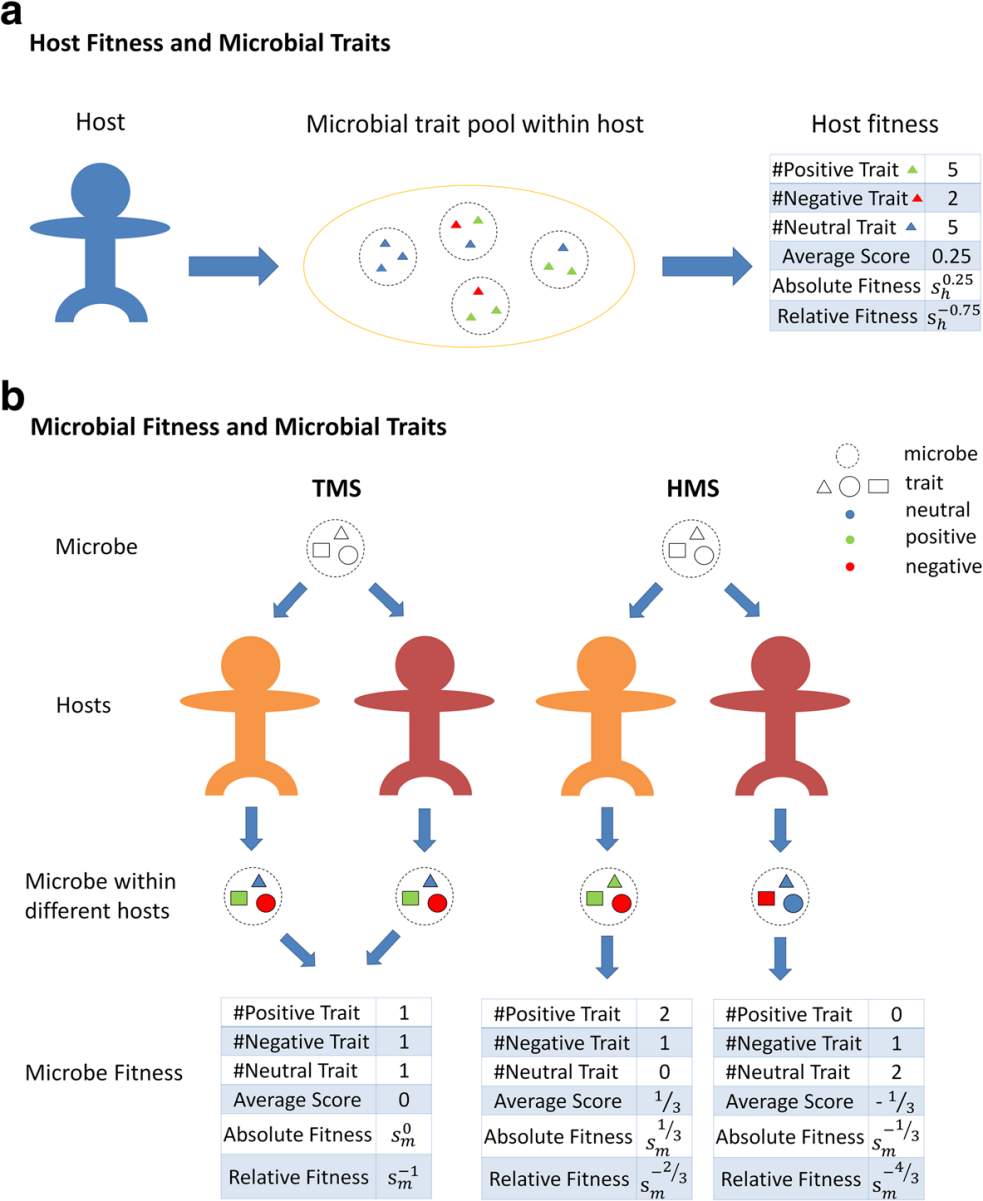
From phenomes to host/microbe fitnesses. The diagram shows how our models connect collections of traits (i.e., phenomes) of microbes to host fitness and microbe fitness. **a** When HS is acting, each trait (represented by a triangle) has a fitness score affecting host fitness (red − 1, green + 1, blue 0). We determined the host absolute fitness and relative fitness with the average fitness score of all microbial traits associated with its microbiome and a host selection coefficient (see “Methods” for details). **b** When TMS/HMS is acting, each trait has another fitness score that affects microbe fitness (red − 1, green + 1, blue 0). The difference between HMS and TMS is that the fitness score of a certain trait is universal for all hosts under TMS but varies from host to host under HMS. Similarly, microbial absolute fitness and relative fitness are also determined with the average fitness score of all microbial traits possessed by the microbe and a microbial selection coefficient (see “Methods” for details)

The neutral models provide a framework which allows the incorporation of other evolutionary and ecological processes. In our current simulations, each microbe has a phenome, consisting of a collection of several traits. By using phenomes, we can incorporate variation in the fitness of microbes, as well as their influence on host fitness, as functions of trait composition. As noted above, this phenome-based approach future-proofs subsequent models in which we expect to take account of trait-by-trait phenomena, including horizontal gene transfer and epistasis. With a phenome-based approach, the selective effects on microbes and hosts are modeled by allowing each trait to confer one fitness value to the microbe itself and, independently, one fitness value to the host where it resides. The two fitness values of a microbial trait can be positive, negative, or neutral and affect the processes of microbiome acquisition and parental assignment, respectively (Figs. 1 and 2).

In our models with selection, microbial taxa are acquired stochastically from the appropriate community (i.e., either the parents or the environment) as a function of their relative abundances within the community and the additive effects of their microbial trait fitness values (Fig. 1b). Similarly, unlike neutral models where microbes do not interfere with host reproductive capacity, parental hosts that have microbiomes with more beneficial microbes have a higher probability of producing offspring, with the probability again determined by the cumulative host fitness values of the microbiome (Fig. 1a). The host fitness values of all traits across all microbial phenomes within an individual host sum to give a total host fitness value for that individual. Once these values are calculated for all hosts, the individual values are normalized such that each host’s reproductive success in the next generation is a function of its fitness value, subject to preserving a constant-sized population. We refer to the selective effects in these simulations as “host selection” (HS).

Microbial fitness values are assigned to traits in two ways. First, we allow the fitness of each microbe to be determined solely by the microbial fitness values assigned to the traits in its phenome. In this scenario, the probability that a microbial OTU is available to offspring or to the environment is a function of the sum of the microbial fitness values, irrespective of the host the microbe is in. We refer to the selective effects in these simulations as trait-mediated microbial selection (TMS).

In a second scenario, each host imposes a different fitness value on a given trait in a phenome. This condition is meant to simulate the condition where differences in host genetics or host microenvironments alter the fitness of microbes with the same trait content. As with other scenarios, the sum of microbial fitness values determines the relative propensity for that microbe to be passed on to the next generation or into the environmental pool. Additionally, however, we allow these host influences to be inherited by the offspring of these hosts in subsequent generations so that offspring also impose the same influences on microbial fitness values. This is equivalent to modeling, say, a host genetic component that preferentially favors certain microbial traits over others and that varies from host to host. We call this host-mediated microbial selection (HMS). In Fig. 2, we illustrate how trait values are assigned, and cumulative fitnesses calculated, under each of the models of selection proposed here.

By simulating microbiome evolution under HS and/or TMS/HMS, we are able to measure a variety of summary statistics, including microbial diversities (*α*-, *β*- and *γ*-diversities which are measured within host, between hosts and within a population, respectively), fitnesses (the means and variances of host fitness and microbe fitnesses), and the association of host and microbial trait fitnesses (measured by the averaged cosine value of the angle between HS or HMS/TMS vectors of trait fitness).

## Results

**Fig. 3:**
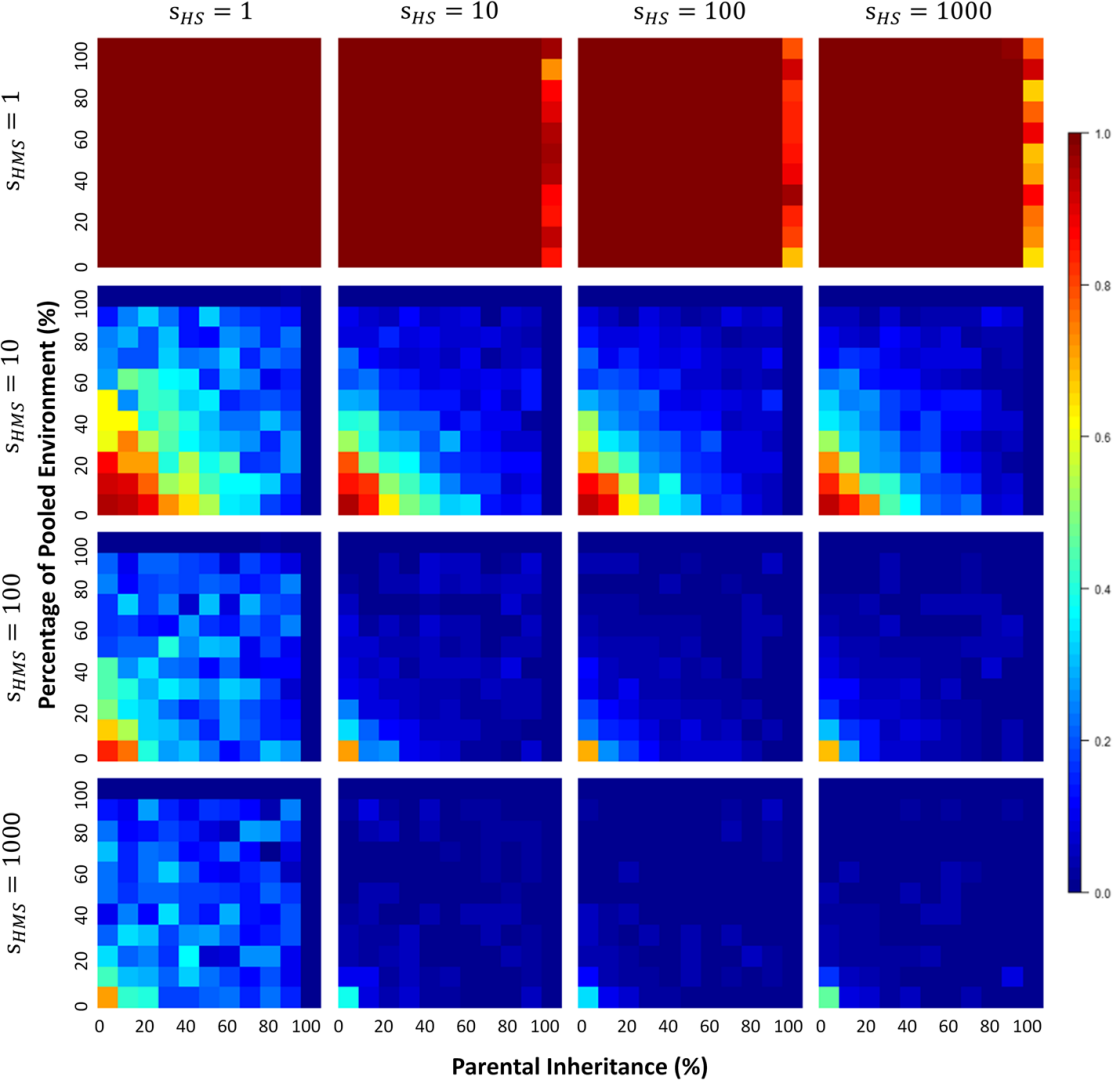
Alpha diversity patterns under models of microbiome evolution with HMS. Each heatmap corresponds to a combination of host and microbial selection parameters both of which have four different levels: 1 (no corresponding selection), 10, 100, and 1000. Microbial selection is implemented as HMS. For each heatmap, horizontal and vertical axes represent percentages of parental contribution and pooled environmental contribution, respectively. The scales of axes are linear with ranges from 0 to 1. The color bar on the right of the heatmaps indicates the corresponding values for diversity (warm color, high diversity; cold color, low diversity)

**Fig. 4:**
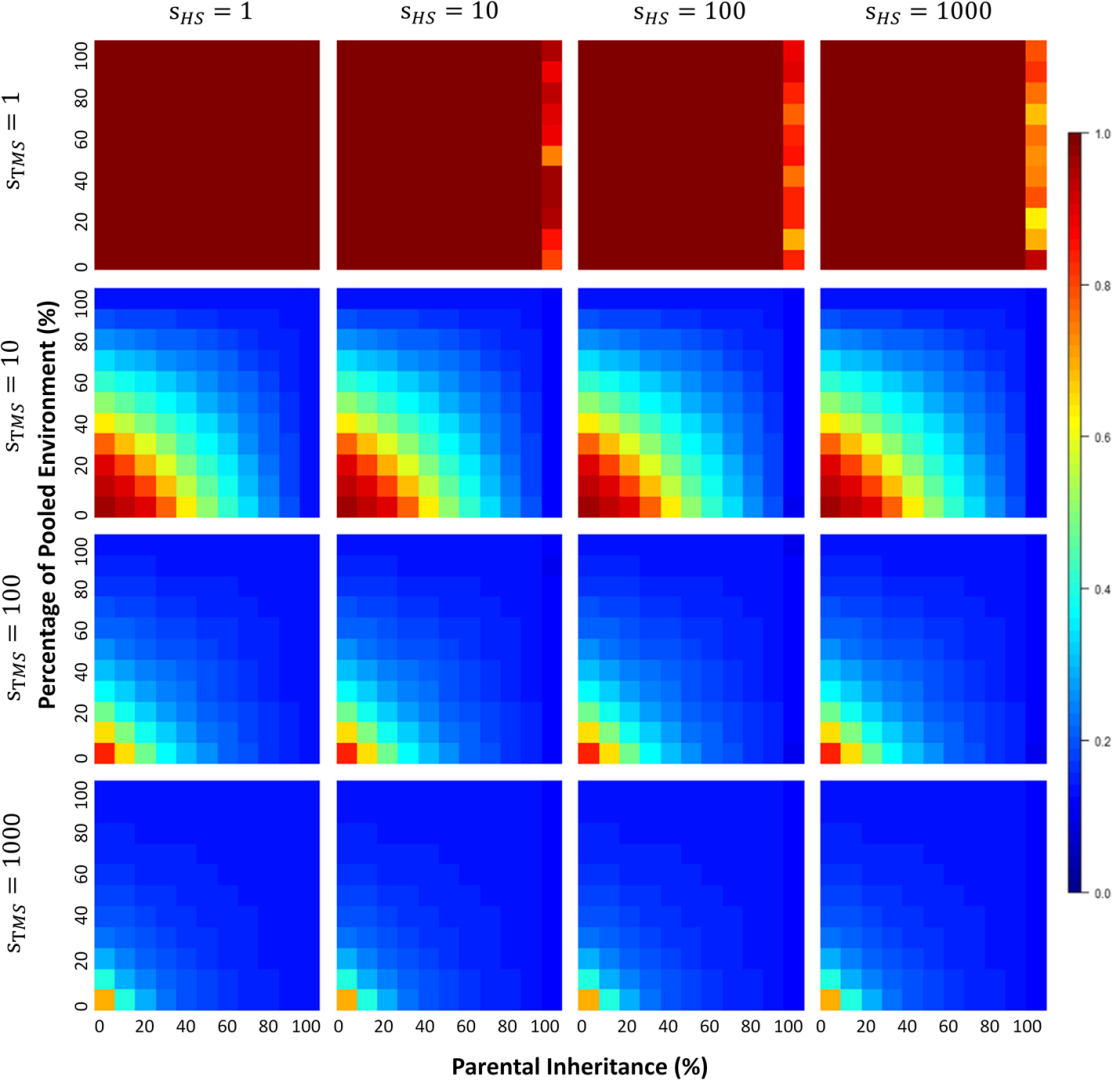
Alpha diversity patterns under models of microbiome evolution with TMS. With a similar layout, all heatmaps are also plotted in the same way as those in Fig. 3 except that microbial selection here is implemented as TMS, not HMS

Our earlier work on neutral models indicated that microbial diversities depend quite strongly on the percentage of parental contribution to offspring microbiomes and the environmental microbial pool [1]. Under neutrality, diversities within hosts remain high, and diversity between hosts remains low for a large part of the range of host contribution. Strong depressive effects on *α*- and *γ*-diversities occur only when host contribution, either directly from parent to offspring or via the environment, is extremely high (> 90%) (Additional file 1: Figure S1). In this paper, when we model these neutral processes with very large numbers of microbes (to 10^9^) and hosts (to 5000), we find no evidence that microbial diversity is depressed under neutral conditions (*s*_*h*_ = *s*_*m*_ = 1) even when parental contributions are high (Figs. 3 and 4). We infer that this is because large numbers of microbes and hosts dampen or completely suppress the stochastic effects of sampling with smaller numbers of microbes and hosts. In other words, when there are many microbes, each microbial OTU in a host will be represented by sufficiently many representatives so that any stochastic effects due to intergenerational transfer of microbes will not remove that OTU from successive descendants.

Under our selective models, microbial diversities are generally lower than those obtained under neutral models across the range of percentages associated with direct parental transmission or parental contributions to the environmental pool (Figs. 3 and 4, Additional file 1: Figure S1 and Additional file 2: Figure S2). Unsurprisingly, increasing the host contribution to offspring microbiomes, either directly through parental transmission or indirectly through contributions to a common environmental pool, can intensify the depressive force of both TMS and HMS on microbial diversities. High host contributions accumulate advantageous microbial taxa and eliminate less fit microbes in source communities, thus leading to microbial diversities that are lower than expected under neutrality.

In contrast to microbial selection, HS alone has almost no effect on microbial diversities, and typically weakly lowers diversities only when host contributions are extremely high (Figs. 3 and 4, Additional file 1: Figure S1 and Additional file 2: Figure S2). This makes sense, of course, when host contributions to subsequent generations are high, microbial taxa act more like inherited traits, and we expect microbes that contribute to host reproductive success to fix in the population in the same way that selectively advantageous traits do (in this case, microbial diversity is loosely analogous to heterozygosity).

There is little difference in the diversity profiles when both HS and TMS are present compared to diversities obtained under TMS alone, thus reinforcing the relatively weak role of HS in shaping microbiome diversities independently (Fig. 4, Additional file 1: Figure S1 and Additional file 2: Figure S2). This is true even when we run our simulations when HS is high (*s*_*h*_ = 1000) (Fig. 4). Interestingly, when both HMS and HS act on the microbial and host populations, we do see microbial diversity decrease as HS increases (Fig. 3), which suggests a strong interaction between HS and HMS.

**Fig. 5:**
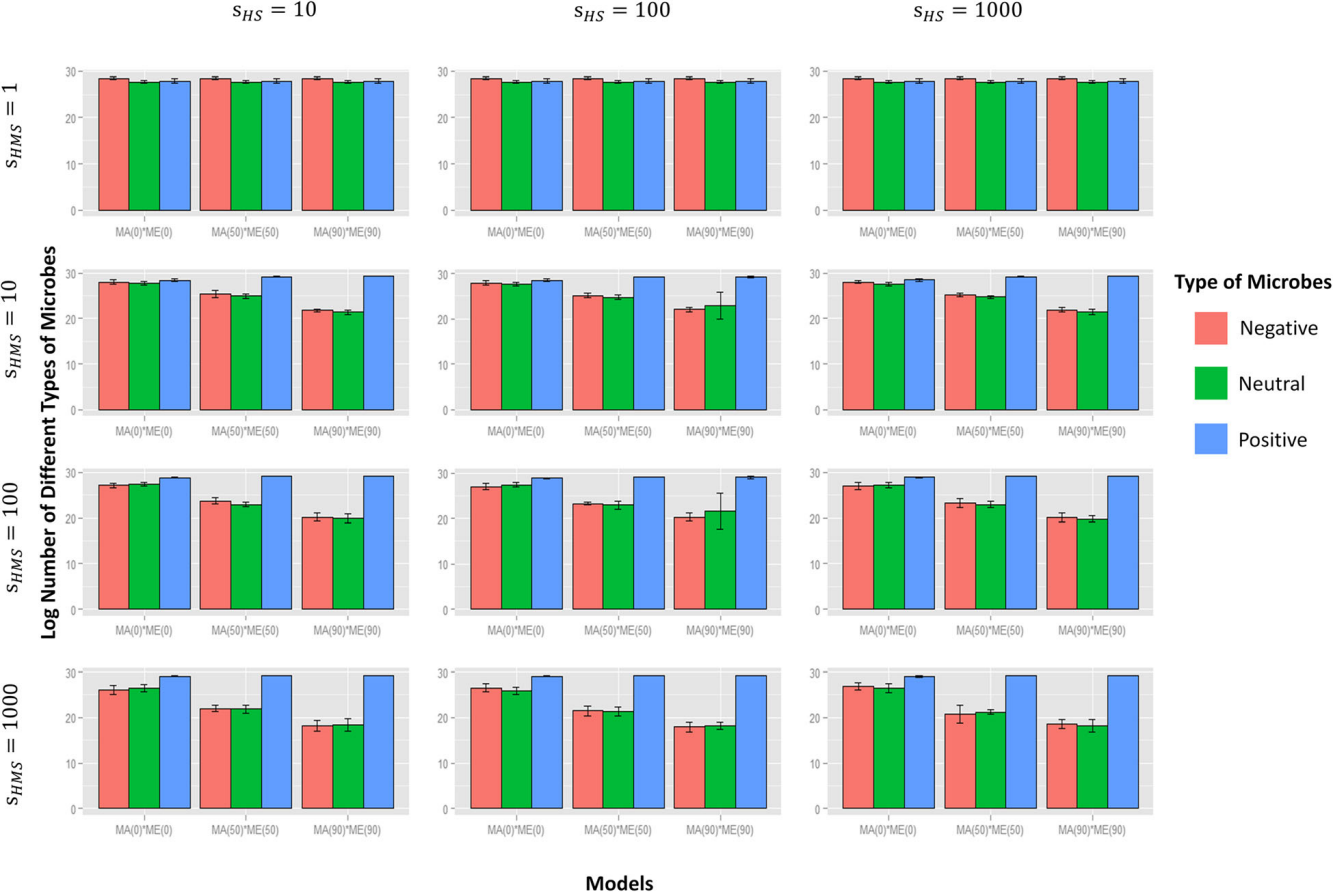
Composition of beneficial, commensal, and pathogenic microbes in host population under different selective models. Each barplot corresponds to a combination of host and microbial selection parameters both of which have four different levels: 1 (no corresponding selection), 10, 100, and 1000. Microbial selection is implemented as HMS. Each bar represents results averaged from five replicate simulations, and color indicates the types of microbes (blue, beneficial; green, commensal; red, pathogenic). Categories on the horizontal axes refer to different combinations of microbiome acquisition and environmental community assembly processes: MA(0)*ME(0) indicates no contributions from parents either directly or to the environment, MA(50)*ME(50) indicates 50% contribution of the parent to the offspring microbiome and 50% of parent to the environment, and MA(90)*ME(90) indicates 90% parental contribution to offspring microbiome and 90% parental contribution to environmental microbial community. Analysis of variance (ANOVA) tests suggest that the compositional changes of three types of microbes are statistically significant as parental contribution and HMS increases (*p* value < 0.0001) but not as HS increases

It is interesting to consider whether OTUs that have a net neutral effect on host fitness (equivalent to “commensal” microbes) and those that have a net negative effect (equivalent to “pathogenic” microbes) persist over the course of the simulations. Since the impact of HS on OTU diversity is high only when HMS is present, we expect to see a proportional increase in beneficial bacteria and an associated decrease in the relative proportion of commensal and pathogenic microbes under this condition. This is indeed what we find (Fig. 5, Additional file 3: Figure S3) with both HS and HMS operating: the proportion of commensal and pathogenic microbes drops, as parental contribution increases. Interestingly, in all simulations, commensals and pathogens are never completely lost from the host population.

**Fig. 6:**
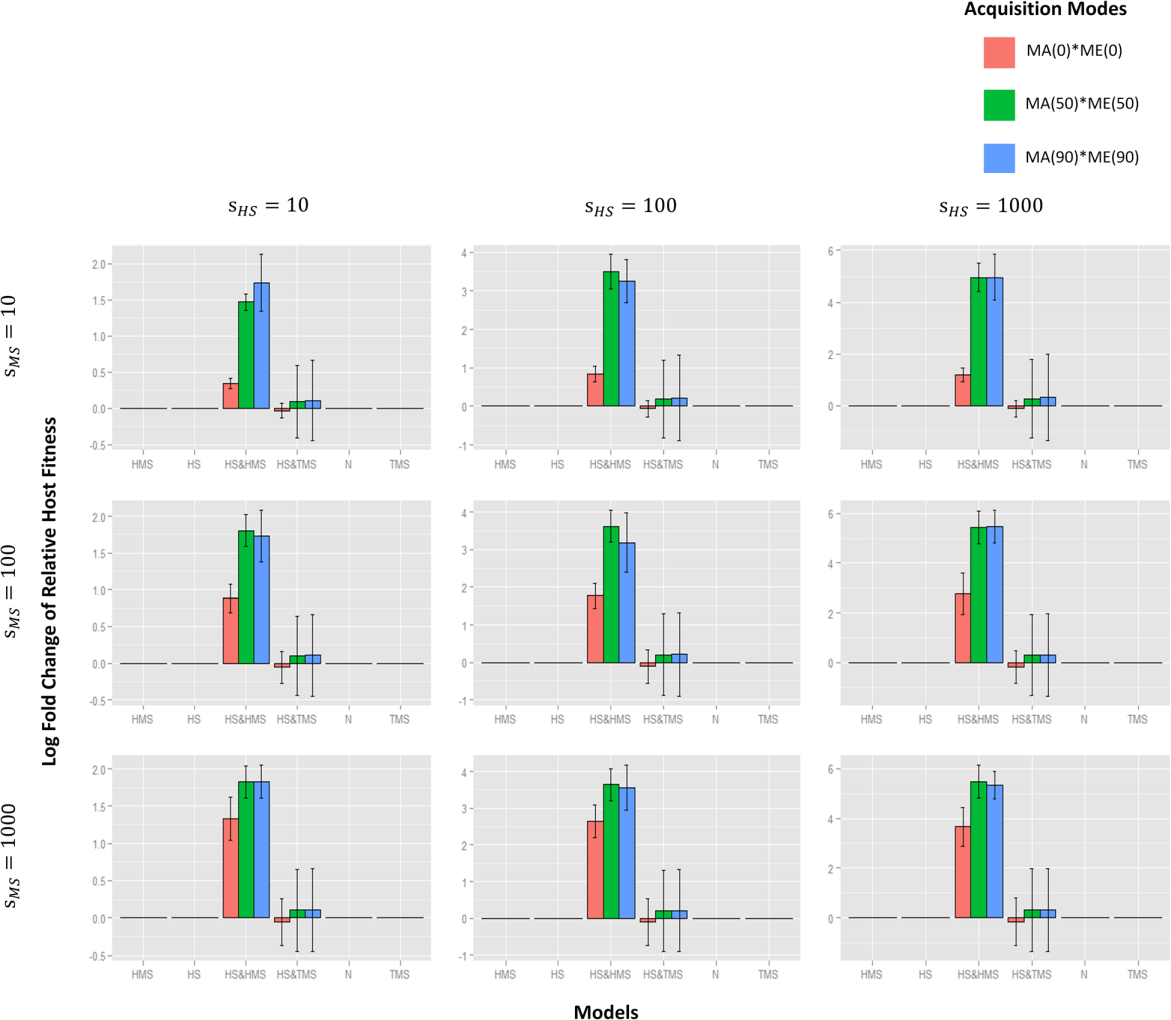
The effects of different models on host fitness. The vertical axis in each figure represents the log final fold change (after 200,000 generations) of average host fitnesses (from five replicates) with respect to the initial levels. The categories on the horizontal axes represent different selective models; colors label different host parental contributions to offspring or environmental microbiomes (see Fig. 5 for description). Each barplot corresponds to a combination of host and microbial selection parameters, both of which have three different levels: 10, 100, and 1000. Host fitness is most strongly affected when HS and HMS apply (even when parental contributions to offspring or environmental microbial communities are absent). Compared with HS and HMS, the improvement of HS alone on host fitness under high parental contribution becomes unobservable

**Fig. 7:**
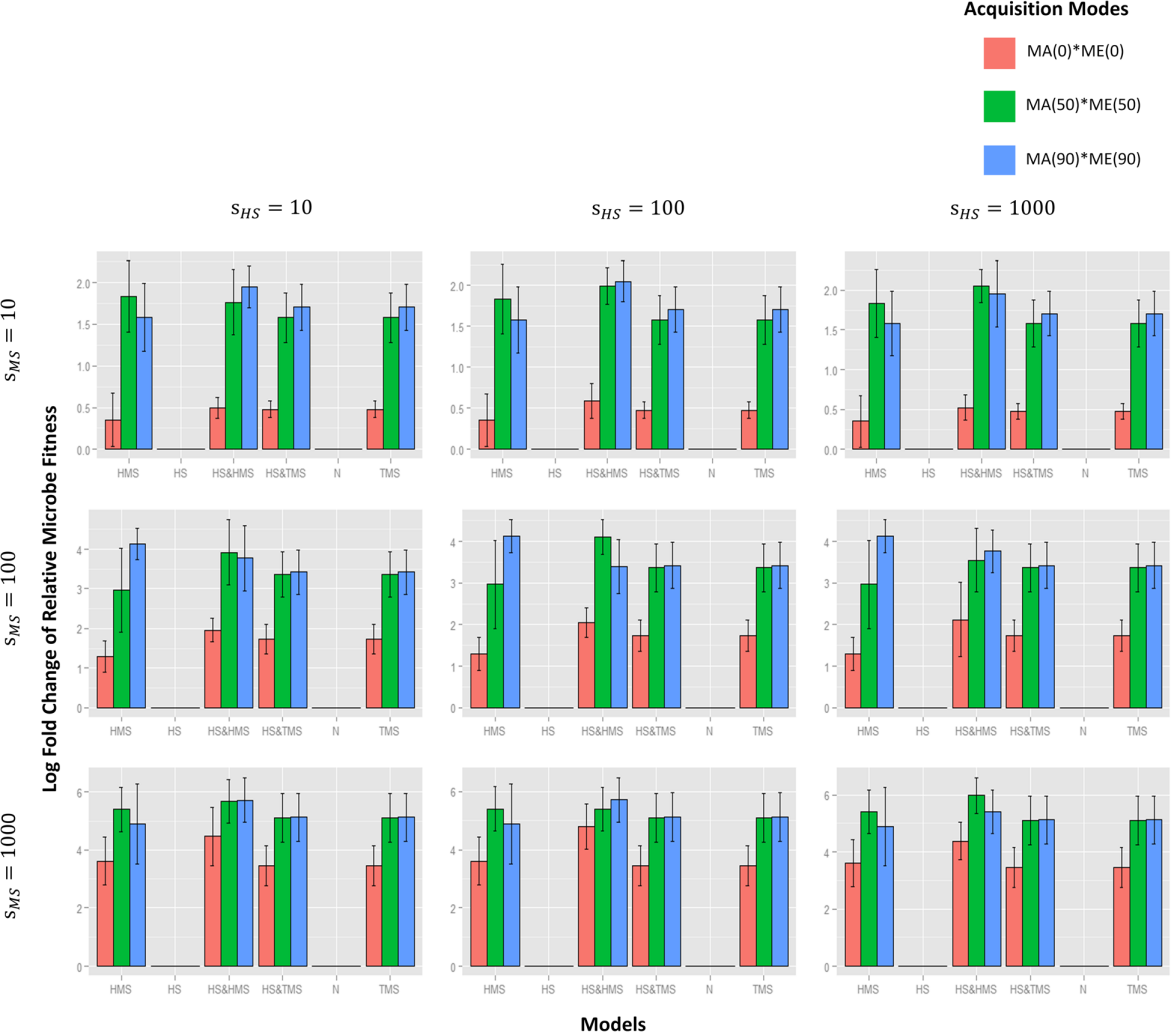
The effects of different models on microbe fitness. With similar layout, barplots are also plotted in the same way as those in Fig. 6 except that the vertical axis represents the log final fold change (after 200,000 generations and from 5 simulations) of average microbe fitnesses (from 5 replicates) with respect to the initial levels. Microbial fitness is strongly influenced by any selective model in which microbial selection operates, i.e., HMS, TMS, HS and HMS, and HS and TMS

Our simulations also allow us to recover the final changes in host and microbial fitnesses with respect to the initial. When only microbial selection operates, either HMS or TMS, average microbial fitness increases but not average host fitness (Figs. 6 and 7, Additional file 4: Figure S4). As with our results on microbial diversity, HS alone improves host fitness (but not microbial fitness) only when direct and/or indirect parental contribution is extremely high (Additional file 4: Figure S4 and Additional file 5: Figure S5).

Interestingly, if HS and TMS are applied together, microbial fitness still increases over time just as with TMS alone (Fig. 7), but changes in host fitness seem to depend on the initial assignment of trait fitness (Additional file 6: Figure S6 and Additional file 7: Figure S7): if, by chance alone, more mutually beneficial traits are assigned at the start of the simulation to phenomes, then host fitness is likely to increase. Conversely, if simulations begin with a predominance of antagonistic trait values (i.e., those in which the selective effects on hosts and microbes work in opposite directions), then mean microbe fitness increases and host fitness decreases. In other words, under HS and TMS, an increase in host fitness is a consequence of an initial “colonization” of microbes that possess traits that are fortuitously advantageous to both hosts and microbes. This is supported by a significantly positive correlation between our measurement of initial HS-TMS trait fitness associations and change in average host fitness (Additional file 6: Figure S6 and Additional file 7: Figure S7).

In contrast, when both HS and HMS operate in a population, both host and microbial fitnesses are improved: host fitness increases more markedly than under HS alone (*p* value < 2.2e−16) and microbial fitness increased more markedly than that under HMS alone (*p* value = 3.215e−07) (Figs. 6 and 7, Additional file 4: Figure S4). This is because hosts that actively acquire and cultivate beneficial microbes that are themselves relatively fitter than other microbes will increase in frequency, thus leading to increasing fitnesses of both hosts and microbes.

## Discussion

In our previous paper [1], we noted that neutral models provide a baseline on which we may build more elaborate models of evolution*.* The adage, attributed to Box [37]*,* that all models are wrong but some are useful, underlies the approach taken by neutral theorists: identify what happens under the simplest processes before adding potentially unnecessary complexity. In this paper, we extend our earlier neutral framework to incorporate selection because there is evidence that neutrality alone cannot explain many of the empirical patterns we observe in microbiomes. Nonetheless, we have tried to stay true to the principle of simplicity, and we have chosen to use selective processes that exclude, for instance, the de novo emergence of host or microbe adaptations. Our models also ignore the fitness of microbes in the environment and their ability to disperse and/or colonize hosts. This is certainly a plausible way of extending the current models; we have chosen not to do so here, simply to avoid the complexity of having to add another fitness vector to our microbial phenomes. Nor have we allowed horizontal gene transfer or microbe-to-microbe interactions. Despite this simplicity, the selective models developed here allow us to study how host and microbial selection affects microbiome diversities and changes in host and microbial fitnesses over time.

First, our simulations indicate that microbiome diversity is more influenced by microbial selection than host selection. In other words, the reproductive success (or otherwise) of hosts that possess certain microbial OTUs does little to shape the overall diversity of host-associated microbial content, unless there is high inheritance of microbes from one generation of hosts to the next. In contrast, when microbes are under selection, mediated either by the host (HMS) or by the traits that they possess (TMS), then fitter microbes are more likely to persist in the microbiome. In other words, our results indicate that microbial selection rather than host selection is a more important determinant of microbial diversities within and between hosts. Biologically, of course, this seems intuitive––we expect to find microbial taxa that are able to live in hosts to be abundantly present within hosts: Bacteroidetes and Firmicutes whose members are able to ferment available nutrients within intestines are the most dominant phylum (more than 98%) in gut communities of more than 60 mammalian species [38, 39]; the low pH environment of the human vagina limits the colonization of other bacteria but allows *Lactobacillus* to thrive (more than 90% of the constituent bacteria in the human vagina are *Lactobacillus* spp) [40, 41]. It is perhaps less intuitive that the benefits that microbial colonizers deliver to hosts (translated into host reproductive success) play a very small role in shaping the eventual diversities of these host-associated microbial communities. In other words, microbiome diversity is not likely to be a consequence of selection acting on hosts; instead, it is most likely to be a consequence of selection acting on microbes.

The exception is when hosts tend to acquire most or all of their microbiomes from their parents or other members within the same population. In these circumstances, beneficial microbes are more likely to be passed on from one generation of hosts to the next, in the same way that selectively advantageous traits sweep through populations. Others have noted that parental and social behaviors stabilize the association between hosts and microbes and increase access to beneficial microbes [42, 43]. For instance, young koalas ingest mothers’ feces to obtain beneficial microorganisms they need for properly digesting eucalyptus leaves [44–46]; the gut microbiota of bumble bees are socially transmitted to protect them against an intestinal parasites [47, 48].

When we consider microbial and host fitnesses, our results are equally illuminating. As offspring acquire a greater proportion of their microbiomes from their parents, microbial fitness increases whenever some microbial selection (either HMS or TMS) is applied (Fig. 7, Additional file 4: Figure S4), although there is no significant difference in microbial fitness outcomes between HMS or TMS. In contrast, the fitness of hosts only increases when both host selection (i.e., HS, selection acting on the fitness of hosts) and host-mediated selection (HMS, selection acting on the microbes) act together (Fig. 6, Additional file 4: Figure S4). Again, this increase appears to depend somewhat on the extent of parental contribution to offspring microbiomes.

Why does this happen? In our model, HMS allows individual hosts to influence microbial fitness by acting directly on their trait values. These microbial fitness values imposed by the hosts are variable and heritable and are equivalent to any host factor that provides an accommodating environment to microbes with the appropriate suite of traits, for example, antigen-specific immunotolerance, microbial “sanctuaries,” or microbial “microenvironments.” Clearly, microbes with the right traits (i.e., in our models, those that possess traits with positive microbial fitness values) in these particular hosts will have higher opportunities to contribute to either the microbiomes of host offspring or to the environmental pool in the succeeding generation. If these microbes have traits that also increase the fitness of particular hosts, then in each successive generation, we expect to see increasing frequencies of these microbes and their hosts over time. This, in turn, will lead to increasing average fitnesses of both hosts and microbes.

This result may resolve an apparent conundrum, alluded to by Rodrigo et al. [49]: how can microbes provide functionally valuable services to hosts, with no obvious mechanisms of transmission and apparently opportunistic acquisition from the environment [50–54]? Arguably, our simulations under HS and HMS capture the dynamics of the emergence of “no-cost” or “byproduct” mutualism between hosts and microbes since there are no active interactions amongst hosts that favor the persistence of beneficial microbes [55, 56].

Of course, when offspring inherit their entire microbiome from their parents, host selective effects can have an effect on host fitness even without the action of HMS on microbes (Additional file 4: Figure S4 and Additional file 5: Figure S5). Here, a large direct or indirect contribution by parents to offspring is analogous to the effect of selection acting on heritable advantageous traits, i.e., we expect that with sufficiently large fitness effects, these will increase in frequency in the population.

Our results also demonstrate that even when both host and microbial selection are in play in a population, microbial communities will continue to harbor microbes that contribute nothing to host fitness (commensals) or contribute negatively (pathogens). The persistence of commensals and pathogens accords with what we expect of host-associated microbial communities. In reality, outbreaks of pathogenic bacteria do occur precisely because these bacteria exist in low levels in the community as a result of opportunistic invasions from environmental reservoirs [57–60]. Under our model, these environmental reservoirs are equivalent to our “fixed environmental component,” in which microbes that have a negative effect on host fitness can persist. As noted by one reviewer, it is also possible that pathogens and commensals may exist because traits in the phenomes of these OTUs also confer, serendipitously, a fitness advantage to survive in the host. Unsurprisingly, the proportion of these “pathogenic” bacteria tends to decrease as the proportion of parental contribution to the environment increases (Fig. 5, Additional file 3: Figure S3).

Our earlier neutral models of microbiome evolution [1] excluded more complex evolutionary processes. The models of selection in this paper add an additional layer of complexity, but they do not encapsulate the complete set of processes that influence microbiome diversities. We have, for instance, excluded mutation and microbial recombination from our models; consequently, host influences and microbial phenomes are static over the time-course of our simulations. Both mutation and recombination are likely to accelerate the rate at which host and microbial fitnesses increase. Similarly, we have excluded population subdivision from structuring our population of hosts. Population subdivision can potentially lead to the maintenance of different communities of microbes in each subpopulation, and subsequent reintroduction of pathogenic or commensal microbes (with migration) into other demes, thus maintaining higher levels of microbial diversity in the larger host population.

To summarize, our results demonstrate that microbial fitness is the primary determinant of microbiome diversity within and between hosts. Host selection plays a significant role in determining microbiome diversity only when direct and/or indirect parental contributions to the microbial communities of offspring are high. We show that regardless of the type of selective effect applied and over a wide range of selective strength, commensal and pathogenic microbes continue to persist in the host population. Finally, we show that even without high parental inheritance, the combination of host selection and host-mediated microbial selection leads to an increase in both host and microbial fitness and the emergence of host-microbe mutualism. Our results explain why opportunistic acquisition of microbes can still deliver functional benefits to hosts.

## Conclusions

Previous studies suggest both hosts and microbes exert these selective filters on their partners, and these filters in turn may influence the evolution of these host-associated microbial communities or microbiomes. Here, we present a computational framework that considered the fitness effects on both host and microbe levels and explore how selection acts to shape microbiomes in a population of hosts. We show that microbiome diversity is most strongly shaped by factors that influence microbial survival and persistence. Our models also demonstrate that selection does not completely deplete the pool of microbes that have no effect on host fitness (i.e., “commensal” microbes) or that have a negative effect on host fitness (i.e., “pathogenic” microbes). Finally, we show that it is possible for bacteria and hosts that mutually sustain each other to dominate microbiomes, even in the absence of high parental contributions from one generation to the next.

## Methods

The simulation details of our neutral models have been described in our previous paper [1]. Again, we employed agent-based forward-time simulations, with discrete host generations, to obtain our results. Under the selective models in this paper, we augment our neutral models by introducing microbial “phenomes.” In our simulations, we applied different combinations of selection and parental contribution parameters to our models.

A constant-sized population of host individuals (*N* = 5000) were simulated with each host individual allocated a microbiome. In our model, the capacity of the microbiome in each host (or the “slots” available for occupation by individual microbes) was fixed as 10^9^ microbes. The initial environmental pool contained 150 microbial OTUs, with each OTU having the same (uniform) abundance. The microbiomes of the initial generation of host individuals were seeded randomly, with microbes sampled from the initial environmental microbial pool. Each microbial OTU was assigned a phenome consisting of five “traits” (*m* = 5) randomly sampled from a uniformly distributed trait pool of 25 available traits (*n* = 25). Phenomes were assigned to each OTU at the beginning of simulation and remained the same throughout the entire simulation (i.e., no changes are made to OTU phenomes through “mutation” or “recombination”). All simulations were run for 200,000 discrete host generations.

Our model parameters are not chosen arbitrarily but set to approximate the real human-associated microbial community as much as possible. We choose 5000 as the host population size because previous population genetic studies estimate the effective population size of humans at ~ 3100 to ~ 10,000 [61–63]. (As a digression, we have chosen to use effective population size in our simulations because the effective size is proportional to the rate of loss/gain of genetic diversity in a population. Although it is outside the scope of this paper, it is interesting to consider whether there is a different host “effective” size that is proportional to the rate of loss/gain of microbiome diversity.) We choose 150 as the total number of OTUs because empirical human microbiome data from the HMP website (http://www.hmpdacc.org/HMSMCP/) suggest that the total number of microbial genera (equivalent to a 97% identity threshold of 16S OTU) associated with different human body sites varies from ~ 50 to ~ 200.

For the total number of available microbial traits, the functional composition of human minimal gut metagenome has been summarized into 25 KEGG functional orthogonal groups [64, 65]. We would like to treat each functional group as one microbial trait under our model, thus 25 can be a reasonable choice of the total number of traits. The distribution of bacteria genome size ranges from 0.13 to 14 Mbp [66–68] with an average of ~ 3 Mbp [69]. Previous studies also suggest a high correlation between bacterial genome size and the number of functional genes and operons given the small amount of non-coding DNA in prokaryotic genomes [70]. If we assume there is a linear correlation between genome size and phenome size and the maximum genome contains all the available traits, the average number of traits across all OTUs can be estimated as:

$$ \mathrm{Average}\  \mathrm{number}\  \mathrm{of}\  \mathrm{traits}\approx \frac{\mathrm{Average}\  \mathrm{genome}\  \mathrm{size}}{\mathrm{Maximum}\  \mathrm{genome}\  \mathrm{size}}\times \mathrm{Total}\  \mathrm{number}\  \mathrm{of}\  \mathrm{traits}=\frac{3\ \mathrm{Mbp}}{14\ \mathrm{Mbp}}\times 25\approx 5 $$

For the size of an individual microbiome, we use 10^9^ to approximate the total number of microbes carried by one host. This huge number may still not be large enough. However, we believe that further increase of the size will not drastically change the currently observed diversity patterns and fitness effects since we also have simulations under much smaller parameter settings that confirm the robustness of our simulated results (Additional file 1: Figure S1, Additional file 2: Figure S2, Additional file 3: Figure S3, Additional file 4: Figure S4 and Additional file 7: Figure S7). For those small-scale simulations, we have host population size of 500, individual microbiome size of 10,000, 150 microbial OTUs, 10 available traits, and 5 traits per OTU. All small-scale simulations were run for 10,000 discrete host generations with 50 replicates.

For a specific OTU, each microbial trait is associated with two numbers (both can be − 1, 0, or 1 independently) to specify a negative, neutral, or positive effect, respectively, on its own fitness and the fitness of the host where it resides. For host selection (HS) and trait-mediated microbial selection (TMS), the assignment of trait fitness was only performed once and applied to all the hosts and microbes. However, for host-mediated microbial selection (HMS), this process was repeated *N* times and each assignment applied uniquely to each host and all its offspring, as well as all the microbes within the host (see Fig. 2). Therefore, for any specific host, all microbes that possess a certain trait still retain the same fitness value for that trait, whereas microbes associated with different hosts can have different fitness values for the same trait.

The probability of sampling microbes from a source community (i.e., either the environmental or the parental microbiomes) is given by:

$$ {p}_i=\frac{f_i{a}_i}{\sum_{k=1}^M{f}_k{a}_k} $$

where *f*_*i*_ represents the fitness of microbial OTU *i* defined by its phenome and the particular model of selection applied, *a*_*i*_ represents the relative abundance of microbial OTU *i* within the source community, and *M* is the total number of microbial OTUs.

The probability that a host individual in a given generation is descended from host *i* in the previous generation is:

$$ {q}_i=\frac{f_i}{\sum_{k=1}^N{f}_k} $$

where *f*_*i*_ represents the fitness of host *i*, and *N* is the host population size.

For any microbe *i* or host *i*, the fitness is given by ${f}_i={s}^{\sum_{j=1}^5{v}_j/5}$, where *v*_*j*_ represents the fitness value of *j*th “trait” for the microbe/host (*j* = 1,…,5), and *s* is a selection parameter (≥ 1) which is related to the strength of selection and can be different for HS and microbial selection. The relative fitness that is generally defined by population geneticists as the survival and/or reproductive rate of microbe *i* or host *i* relative to the maximum survival can be further derived as *w*_*i*_ = *f*_*i*_/*s*. In our plots of host and microbe fitnesses, we used *w*_*i*_ to represent fitness (Figs. 6 and 7, Additional file 1: Figure S1 and Additional file 6: Figure S6). When *s*_*h*_ = *s*_*m*_ = 1, *f* and *w* are always equal to 1, and they are equivalent with the neutral models. For our simulations, we simulate with different combinations of *s*_*m*_ and *s*_*h*_ with  *s*_*m*_ ∈ {1,10,100,1000} and *s*_*h*_ ∈ {1,10,100,1000} , which means after one single host generation, the most competitive microbe/host is expected to produce ${s}_m^2$ or ${s}_h^2$ times as many offspring as the least competitive microbe/host does.

The definition of *f*_*i*_ under our model can be derived from a simplified form of the Lotka–Volterra equation without consideration of species interactions.

$$ \frac{d{x}_i(t)}{dt}={\alpha}_i{x}_i(t) $$

where *x*_*i*_(*t*) is the abundance of OTU *i* at time *t* and *α*_*i*_ is its specific growth rate.

The integration of the differential equation above gives us: ${x}_i\left(t+\Delta  t\right)={x}_i(t){e}^{\alpha_i\Delta  t}$. Let ∆*t* be one host generation time *T*, then ${x}_i\left(t+T\right)={x}_i(t){e}^{\alpha_iT}$.

If all microbial species has the same growth rate, ${e}^{\alpha_iT}$ term is the same for all microbial taxa. Thus, after one host generation, the acquired microbial relative abundances will only be proportional to the initial relative abundances *x*_*i*_(*t*) which can be seen as the relative abundances in the source community. This is equivalent to our neutral assembly process.

Under our selective model, if we allow the specific growth rate *α*_*i*_ ∈ [−*a*, *a*] (*a* ≥ 0), we have

$$ {x}_i\left(t+T\right)={x}_i(t){e}^{\alpha_iT}={x}_i(t){\left({e}^{aT}\right)}^{\alpha_i/a} $$

Let *s* = *e* ^*aT*^ and *β*_*i*_ = *α*_*i*_/*a*, we have ${x}_i\left(t+T\right)={x}_i(t){s}^{\beta_i}$ where *β*_*i*_ ∈ [−1, 1] and *s* ≥ 1. Under our model, *s* becomes the selection coefficient we defined and ${\beta}_i=\sum_{j=1}^m{v}_j/m$ is represented as the average fitness score contributed by all the microbial traits. Thus, after one host generation, the acquired microbial relative abundances will be proportional to the product of the initial relative abundance *x*_*i*_(*t*) and the corresponding microbial fitness term ${s}^{\beta_i}$. As mentioned before, our microbial selection coefficient can be interpreted as follows: after a single host generation, the most competitive microbe is expected to produce ${s}_m^2$ times as many offspring as the least competitive microbe does. Now, for *s* = *e* ^*aT*^, both *e* and *T* are constant and only *a* is a variable; so, we can also interpret it in this way: after a single host generation, the most competitive microbial taxon has a specific growth rate *a* while the least competitive microbial taxon has a specific growth rate − *a* where $a=\frac{\ln (s)}{T}$. Similarly, host fitness can be derived in the same way.

As mentioned above, the microbiome of each host is associated with two fitness vectors with size equal to the total number of traits in the population (i.e., 10 traits), as depicted in Fig. 2. The correlation of these fitness vectors under HS and TMS/HMS is defined as the cosine of the angle between them and is calculated as:

$$ \cos \theta =\frac{\sum_{k=1}^{10}{h}_k{m}_k}{\sqrt{\sum_{k=1}^{10}{h}_k^2\sum_{k=1}^{10}{m}_k^2}} $$

where *h*_*k*_ and *m*_*k*_ represent the host and microbial fitness values, respectively, of *k*th microbial trait (*k* = 1,…, 10). At the population level, we take the average of each host’s cos*θ* to calculate the consistency. Biologically, a positive consistency means HS and MS generally favor the same microbial genes while a negative consistency means microbial genes favored HS/MS are generally disfavored by MS/HS. Zero consistency simply means one type of selection exerts no influence on those microbial genes that are positively or negatively selected by the other.

Three microbial diversities are measured in the same way as previously described [1]: *α*- and *γ*-diversities are measured with Shannon-Wiener index, and *β*-diversity is measured with Bray-Curtis distance.

All statistical tests and distribution/model fitting were conducted using the R software package [71]. For Figures S1 and S7, a test for Pearson product-moment correlation was performed to identify the association between the initial HS-TMS consistency and change in host fitness. For Fig. 5, an ANOVA test was applied to evaluate the statistical significance of microbial compositional change as parental contribution, HMS, and HS increase. For Figs. 6 and 7, a paired two-sample Wilcoxon test was conducted to confirm the promotive effects of HS and HMS on fitnesses compared to HS or HMS alone. For Additional file 6: Figure S6, a Student’s *t* test was performed to examine the effects of different selective models on fitnesses against the null neutral models.

Each combination of parameters was run for 5 simulations, except for small-scale simulations (with 50 replicates) and simulations reported in Additional file 2: Figure S2, which was run for 1,000,000 generations without any replicates. All simulations were carried out using Java programs, available from https://github.com/qz28/microbiosima.git.

## Additional files

Additional files 1: Figure S1. Diversity patterns under different models of microbiome selection. This plot is based on small-scale simulations. The first row represents results obtained under our neutral model [1]. Rows (b)–(f) represent diversities obtained under models of selection (b TMS, c HMS, d HS, e HS and TMS; f, HS and HMS). Heatmaps in each column display measurements of different diversity measures, from left to right, *α*-diversity, *γ*-diversity, and *β*-diversity. For each heatmap, horizontal and vertical axes represent percentages of parental contribution and pooled environmental contribution, respectively. Under neutral and HS models, the scales of axes are non-positive exponentials of 2 with ranges from 0 approaching to 1. For heatmaps in (b), (c), (e), and (f), the scales of axes are linear with ranges from 0 to 1. The color bar on the right of the heatmaps indicates the corresponding values for diversity (warm color, high diversity; cold color, low diversity). (TIFF 2395 kb)

Additional files 2: Figure S2. Diversity patterns under different models of microbiome selection. This plot is based on small-scale simulations. The first row represents results obtained under our neutral model [1]. Rows (b)–(f) represent diversities obtained under models of selection (b TMS, c HMS, d HS, e HS and TMS; f, HS and HMS). Heatmaps in each column display measurements of different diversity measures, from left to right, *α*-diversity, *γ*-diversity, and *β*-diversity. For each heatmap, horizontal and vertical axes represent percentages of parental contribution and pooled environmental contribution, respectively. Under neutral and HS models, the scales of axes are non-positive exponentials of 2 with ranges from 0 approaching to 1. For heatmaps in (b), (c), (e), and (f), the scales of axes are linear with ranges from 0 to 1. The color bar on the right of the heatmaps indicates the corresponding values for diversity (warm color, high diversity; cold color, low diversity). (TIFF 2293 kb)

Additional files 3: Figure S3. Composition of beneficial, commensal, and pathogenic microbes in host population under different selective models. This plot is based on small-scale simulations. Stacked barplot labeled with “initial” shows the initial composition of these three types of microbes in host population; all simulations start with the same initial conditions. Stacked barplot labeled with “HS,” “HS and TMS,” and “HS and HMS” shows the ultimate composition of these three type of microbes in host populations under the respective selective model. Each bar represents results averaged from 50 replicate simulations, and gray scale indicates the types of microbes (white, beneficial; gray, commensal; black, pathogenic). Categories on the horizontal axes refer to different combinations of microbiome acquisition and environmental community assembly processes: MA(0)*ME(0) indicates no contributions from parents either directly or to the environment, MA(50)*ME(50) indicates 50% contribution of the parent to the offspring microbiome and 50% of parent to the environment, and MA(90)*ME(90) indicates 90% parental contribution to offspring microbiome and 90% parental contribution to environmental microbial community. (TIFF 1293 kb)

Additional files 4: Figure S4. The effects of different models on microbe fitness and host fitness. This plot is based on small-scale simulations. Panels (a) and (b) show the effects of different models of selection on final values of average microbial fitness and host fitness, respectively. The vertical axis in each figure represents the final fold change of average microbe or host fitnesses with respect to the initial levels. The categories on the horizontal axes represent different selective models; colors ranging from black to white label different host parental contributions to offspring or environmental microbiomes (see Fig. 4 for description). Over each bar, asterisks indicate the statistical significance of differences from neutral models (****p value < 0.0001, ***p value < 0.001, **p value < 0.01, *p value < 0.05). For panel (a), microbial fitness is strongly influenced by any selective model in which microbial selection operates, i.e., HMS, TMS, HS and HMS, and HS and TMS. For panel (b), host fitness is most strongly affected when HS and HMS apply (even when parental contributions to offspring or environmental microbial communities are absent) or when HS or HS and TMS operate with high parental contributions. (TIFF 4270 kb)

Additional files 5: Figure S5. The effects of HS on microbe and host fitnesses under pure parental acquisition. Each point corresponds to one simulation implemented under HS alone and pure parental acquisition for 1000,000 host generations. The vertical axes represent the log final fold change of average host/microbe fitnesses with respect to the initial levels (red dot, host fitness; blue triangle, microbe fitness). The horizontal axes represent the logarithm of host selection parameters with respect to base 10. (TIFF 710 kb)

Additional files 6: Figure S1. Scatterplots of host fitness changes and initial HS-TMS consistency under host and microbial selections. Each dot represents one simulation performed under the selective model of HS and TMS (*s*_*HS*_ *= s*_*TMS*_ *=* 10). For each simulation, the HS-TMS consistency is a fixed value over time but randomly initialized since the trait fitnesses to host and microbe are randomly assigned at the very beginning. The changes in average fitness of host population is measured by subtracting the initial fitness level from the final (positive value means increased fitness and negative value means decreased fitness). Pearson correlation tests were performed and suggested a strongly positive correlation (*r* = 0.6413382) between host fitness changes and initial HS-TMS consistency (*p* value < 2e^−16). (TIFF 300 kb)

Additional files 7: Figure S7. A scatterplot for visualizing the positively correlated relationship between host fitness changes and initial HS-TMS consistency. This plot is based on small-scale simulations. Each dot represents one simulation performed under the selective model of HS and TMS. For each simulation, the HS-TMS consistency is a fixed value over time but randomly initialized since the trait fitnesses to host and microbe are randomly assigned at the very beginning. The changes in average fitness of the host population is measured by subtracting the initial fitness level from the final (positive value means increased fitness and negative value means decreased fitness). (TIFF 2927 kb)

## Abbreviations

HMS: Host-mediated microbial selection
HS: Host selection
MA(x): Mixed acquisition with *x*% from parental inheritance and (1 − *x*)% from environment
ME(y): Mixed environmental microbiome with *y*% from pooled environment and (1 − *y*)% from fixed environment
MS: Microbial selection including HMS and TMS
TMS: Trait-mediated microbial selection
