# Supplementary figures and images for: Models of microbiome evolution incorporating host and microbial selection
Source: Microbiome. 2017 Sep 25;5:127. doi: 10.1186/s40168-017-0343-x (PMC5613328; doi:10.1186/s40168-017-0343-x)

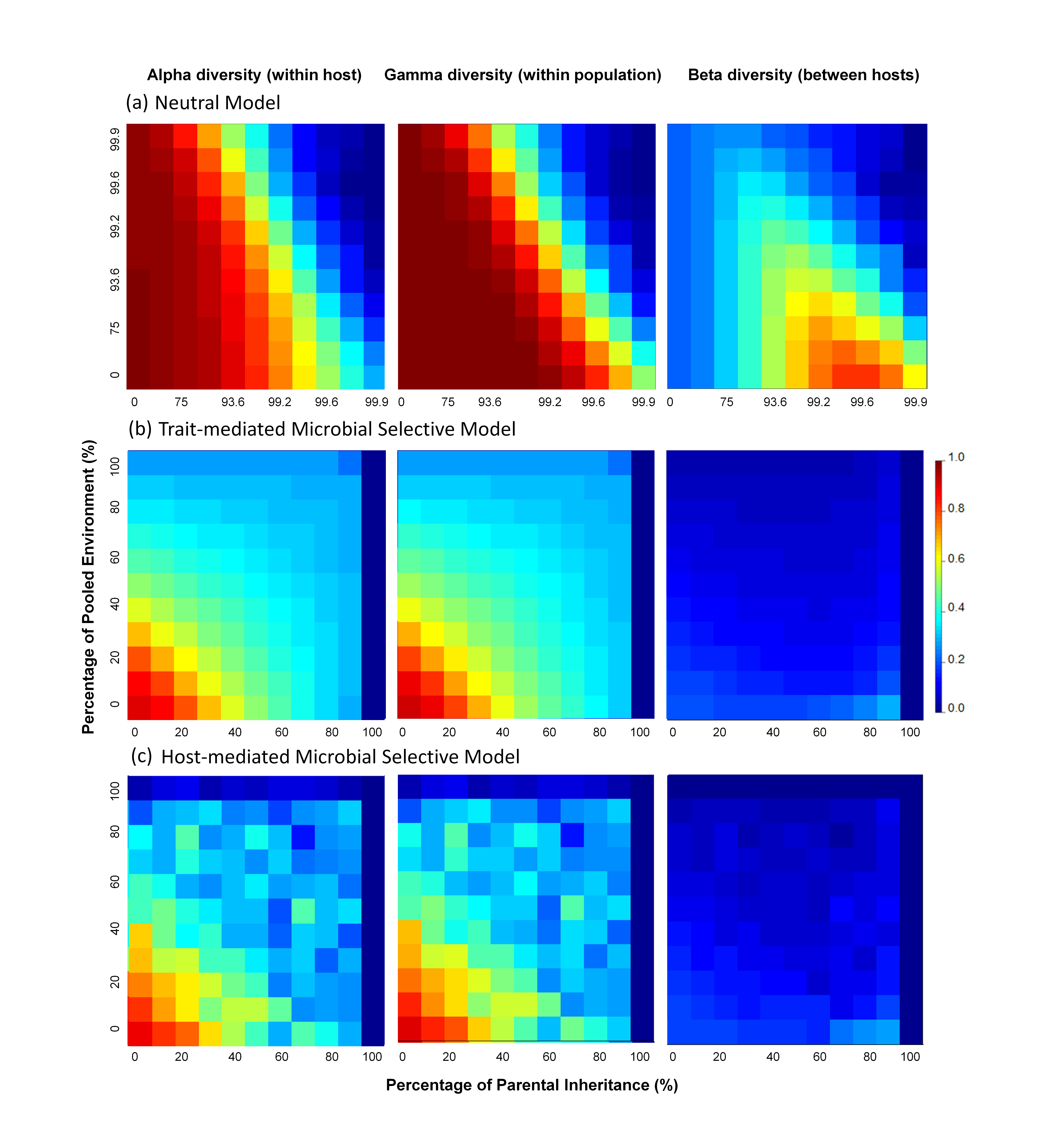

Supplement: Supplementary file 1 — Diversity patterns under different models of microbiome selection. This plot is based on small-scale simulations. The first row represents results obtained under our neutral model [1]. Rows (b)–(f) represent diversities obtained under models of selection (b TMS, c HMS, d HS, e HS and TMS; f, HS and HMS). Heatmaps in each column display measurements of different diversity measures, from left to right, α-diversity, γ-diversity, and β-diversity. For each heatmap, horizontal and vertical axes represent percentages of parental contribution and pooled environmental contribution, respectively. Under neutral and HS models, the scales of axes are non-positive exponentials of 2 with ranges from 0 approaching to 1. For heatmaps in (b), (c), (e), and (f), the scales of axes are linear with ranges from 0 to 1. The color bar on the right of the heatmaps indicates the corresponding values for diversity (warm color, high diversity; cold color, low diversity). (TIFF 2395 kb) [file 40168_2017_343_MOESM1_ESM.tif]

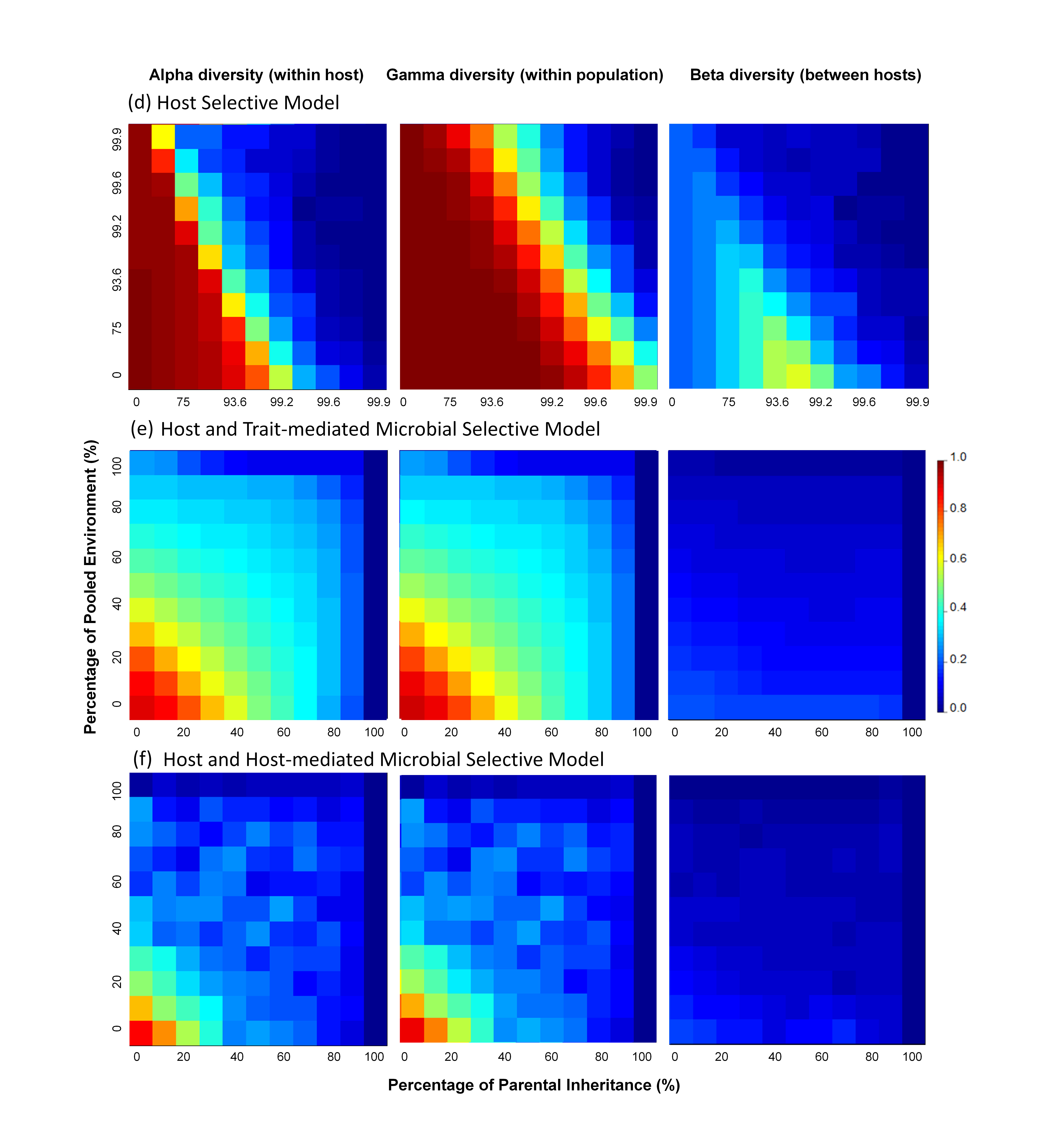

Supplement: Supplementary file 2 — Diversity patterns under different models of microbiome selection. This plot is based on small-scale simulations. The first row represents results obtained under our neutral model [1]. Rows (b)–(f) represent diversities obtained under models of selection (b TMS, c HMS, d HS, e HS and TMS; f, HS and HMS). Heatmaps in each column display measurements of different diversity measures, from left to right, α-diversity, γ-diversity, and β-diversity. For each heatmap, horizontal and vertical axes represent percentages of parental contribution and pooled environmental contribution, respectively. Under neutral and HS models, the scales of axes are non-positive exponentials of 2 with ranges from 0 approaching to 1. For heatmaps in (b), (c), (e), and (f), the scales of axes are linear with ranges from 0 to 1. The color bar on the right of the heatmaps indicates the corresponding values for diversity (warm color, high diversity; cold color, low diversity). (TIFF 2293 kb) [file 40168_2017_343_MOESM2_ESM.tif]

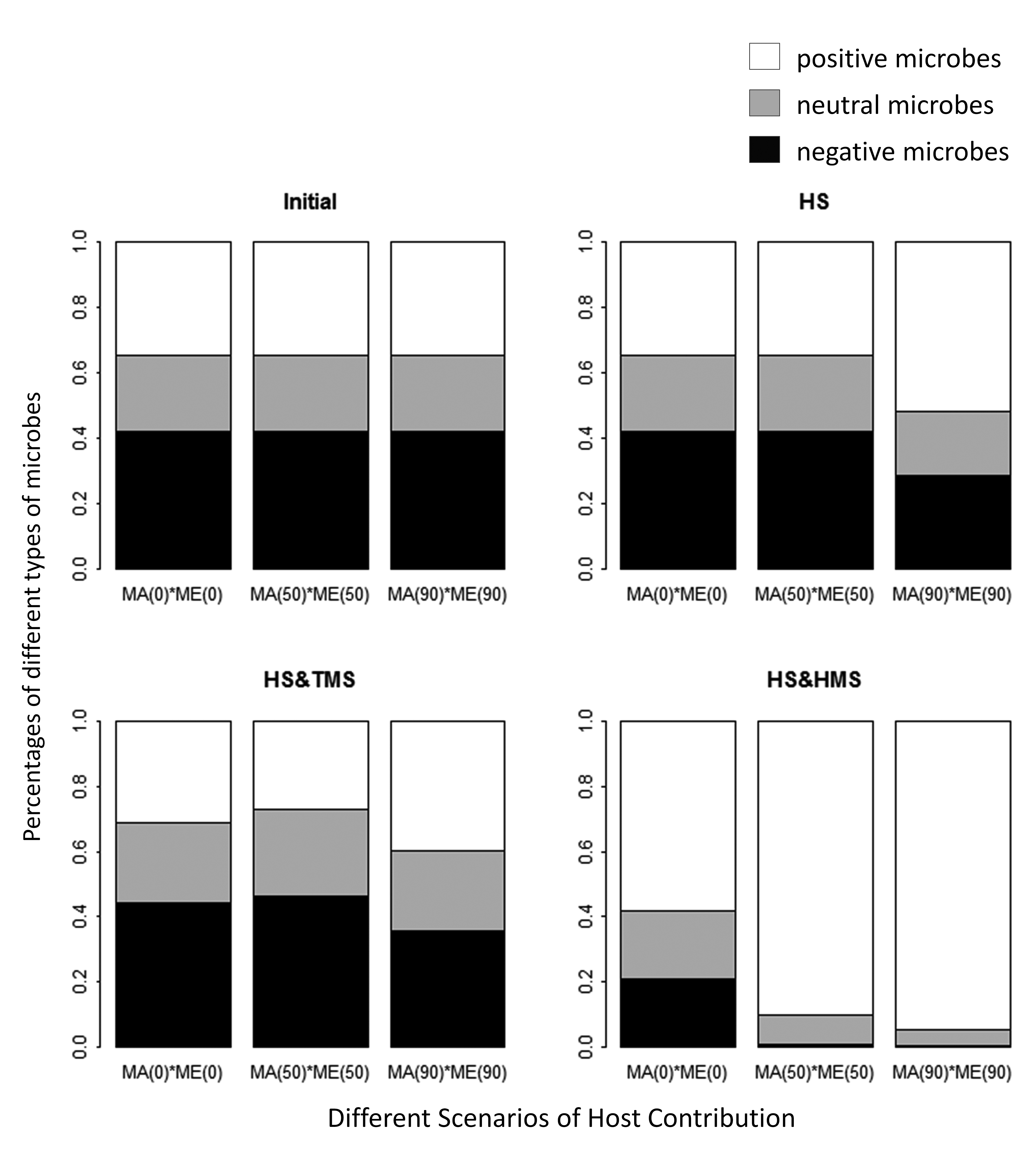

Supplement: Supplementary file 3 — Composition of beneficial, commensal, and pathogenic microbes in host population under different selective models. This plot is based on small-scale simulations. Stacked barplot labeled with “initial” shows the initial composition of these three types of microbes in host population; all simulations start with the same initial conditions. Stacked barplot labeled with “HS,” “HS and TMS,” and “HS and HMS” shows the ultimate composition of these three type of microbes in host populations under the respective selective model. Each bar represents results averaged from 50 replicate simulations, and gray scale indicates the types of microbes (white, beneficial; gray, commensal; black, pathogenic). Categories on the horizontal axes refer to different combinations of microbiome acquisition and environmental community assembly processes: MA(0)*ME(0) indicates no contributions from parents either directly or to the environment, MA(50)*ME(50) indicates 50% contribution of the parent to the offspring microbiome and 50% of parent to the environment, and MA(90)*ME(90) indicates 90% parental contribution to offspring microbiome and 90% parental contribution to environmental microbial community. (TIFF 1293 kb) [file 40168_2017_343_MOESM3_ESM.tif]

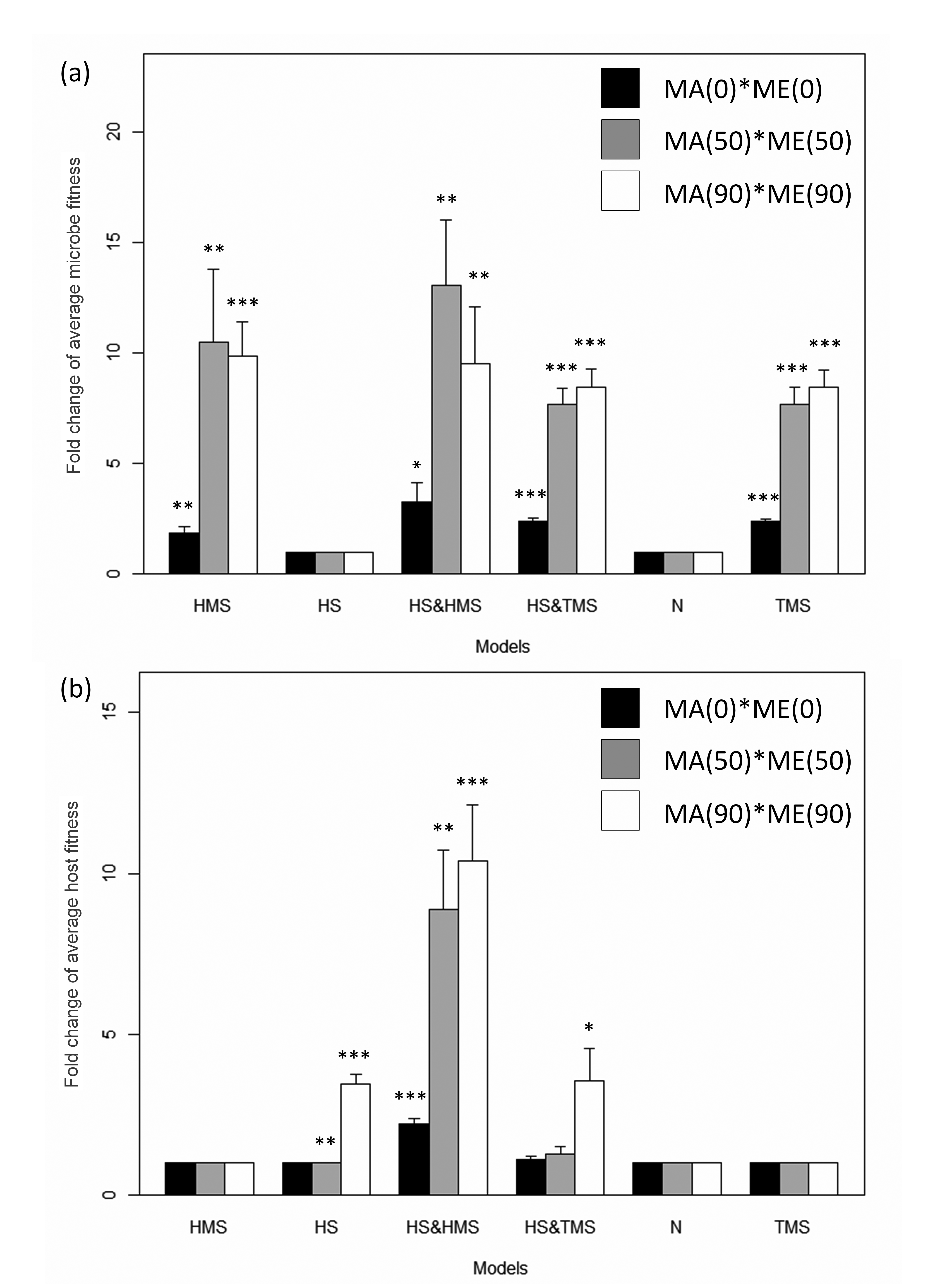

Supplement: Supplementary file 4 — The effects of different models on microbe fitness and host fitness. This plot is based on small-scale simulations. Panels (a) and (b) show the effects of different models of selection on final values of average microbial fitness and host fitness, respectively. The vertical axis in each figure represents the final fold change of average microbe or host fitnesses with respect to the initial levels. The categories on the horizontal axes represent different selective models; colors ranging from black to white label different host parental contributions to offspring or environmental microbiomes (see Fig. 4 for description). Over each bar, asterisks indicate the statistical significance of differences from neutral models (****p value < 0.0001, ***p value < 0.001, **p value < 0.01, *p value < 0.05). For panel (a), microbial fitness is strongly influenced by any selective model in which microbial selection operates, i.e., HMS, TMS, HS and HMS, and HS and TMS. For panel (b), host fitness is most strongly affected when HS and HMS apply (even when parental contributions to offspring or environmental microbial communities are absent) or when HS or HS and TMS operate with high parental contributions. (TIFF 4270 kb) [file 40168_2017_343_MOESM4_ESM.tif]

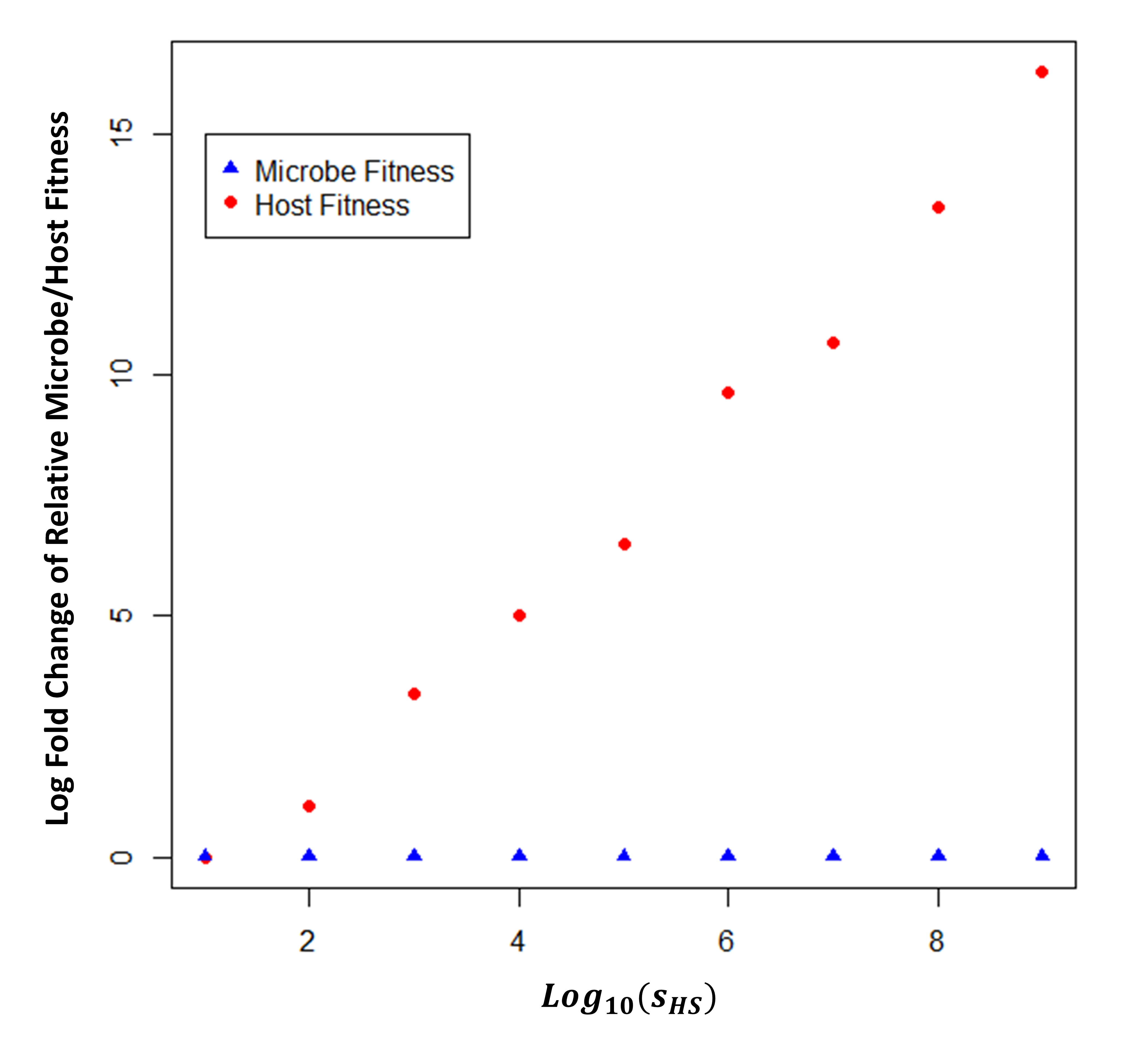

Supplement: Supplementary file 5 — The effects of HS on microbe and host fitnesses under pure parental acquisition. Each point corresponds to one simulation implemented under HS alone and pure parental acquisition for 1000,000 host generations. The vertical axes represent the log final fold change of average host/microbe fitnesses with respect to the initial levels (red dot, host fitness; blue triangle, microbe fitness). The horizontal axes represent the logarithm of host selection parameters with respect to base 10. (TIFF 710 kb) [file 40168_2017_343_MOESM5_ESM.tif]

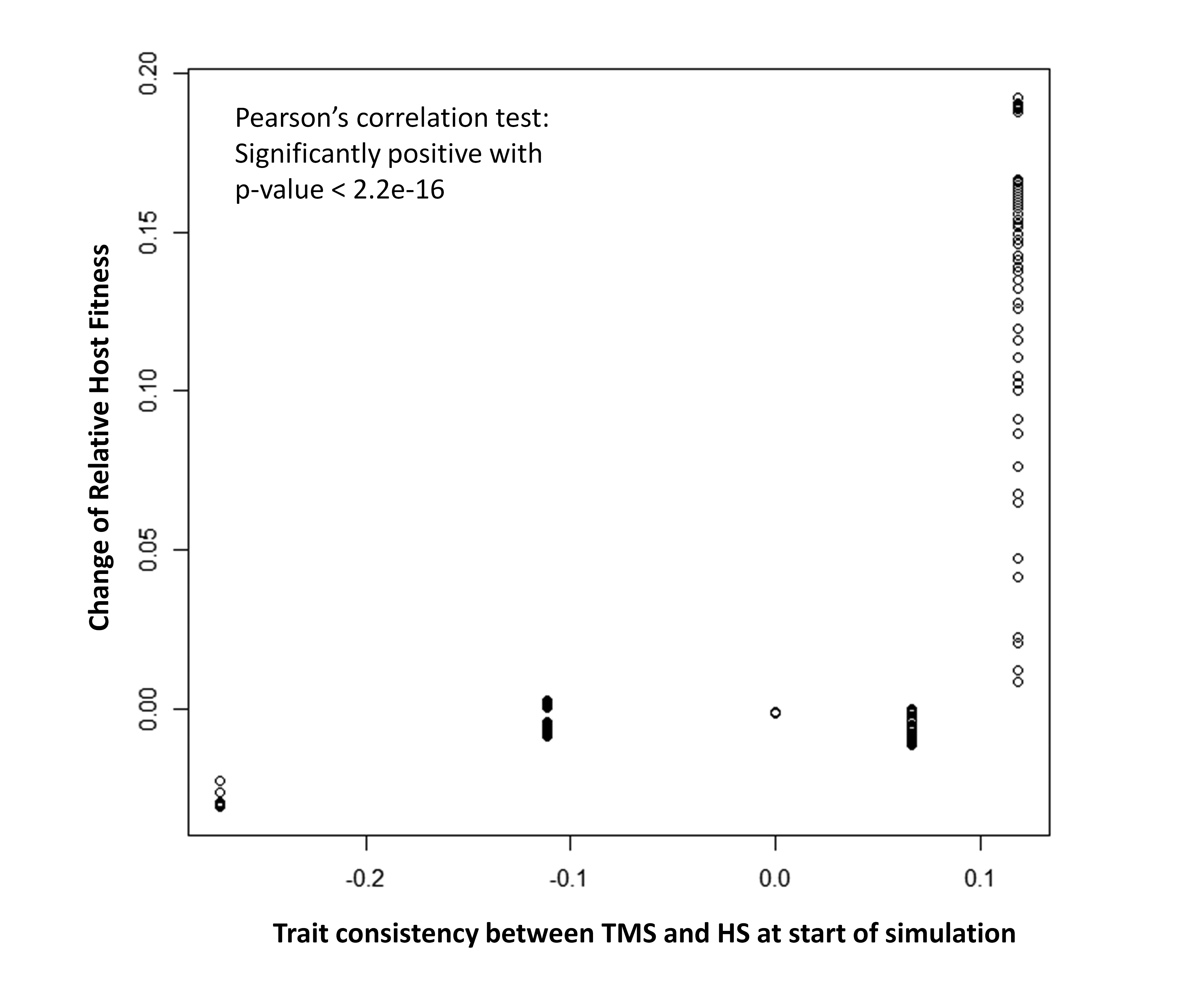

Supplement: Supplementary file 6 — Scatterplots of host fitness changes and initial HS-TMS consistency under host and microbial selections. Each dot represents one simulation performed under the selective model of HS and TMS (s HS = s TMS = 10). For each simulation, the HS-TMS consistency is a fixed value over time but randomly initialized since the trait fitnesses to host and microbe are randomly assigned at the very beginning. The changes in average fitness of host population is measured by subtracting the initial fitness level from the final (positive value means increased fitness and negative value means decreased fitness). Pearson correlation tests were performed and suggested a strongly positive correlation (r = 0.6413382) between host fitness changes and initial HS-TMS consistency (p value < 2e^−16). (TIFF 300 kb) [file 40168_2017_343_MOESM6_ESM.tiff]

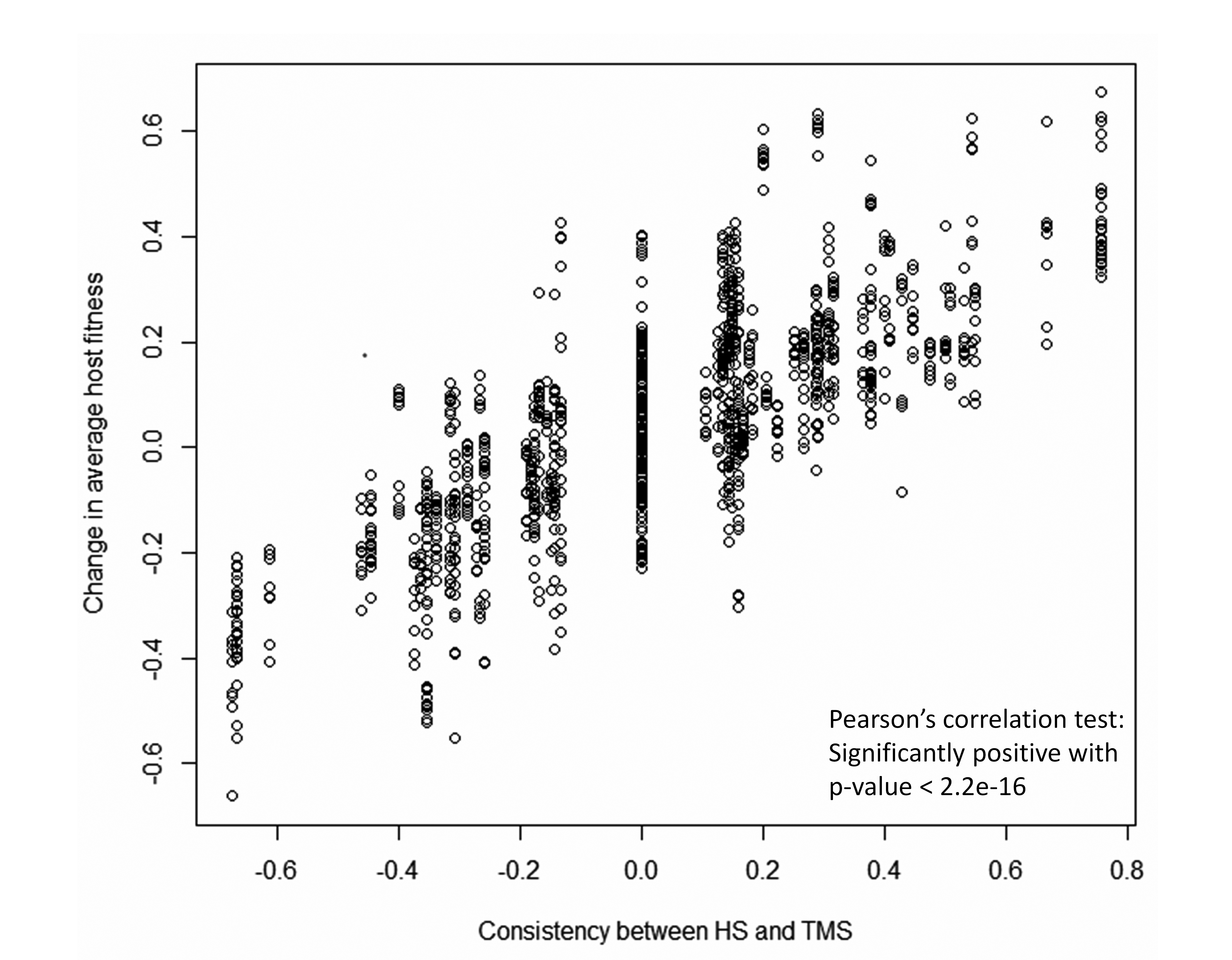

Supplement: Supplementary file 7 — A scatterplot for visualizing the positively correlated relationship between host fitness changes and initial HS-TMS consistency. This plot is based on small-scale simulations. Each dot represents one simulation performed under the selective model of HS and TMS. For each simulation, the HS-TMS consistency is a fixed value over time but randomly initialized since the trait fitnesses to host and microbe are randomly assigned at the very beginning. The changes in average fitness of the host population is measured by subtracting the initial fitness level from the final (positive value means increased fitness and negative value means decreased fitness). (TIFF 2927 kb) [file 40168_2017_343_MOESM7_ESM.tif]
